# Heavy Metal Interactions with Neuroglia and Gut Microbiota: Implications for Huntington’s Disease

**DOI:** 10.3390/cells13131144

**Published:** 2024-07-03

**Authors:** Yousef Tizabi, Samia Bennani, Nacer El Kouhen, Bruk Getachew, Michael Aschner

**Affiliations:** 1 Department of Pharmacology, Howard University College of Medicine, Washington, DC 20059, USA; 2 Faculty of Medicine and Pharmacy of Casablanca, Hassan II University, Casablanca 20670, Morocco; 3 Department of Molecular Pharmacology, Albert Einstein College of Medicine, Bronx, NY 10461, USA

**Keywords:** Huntington’s disease, heavy metals, iron, manganese, copper, glial cells, gut microbiota, neuroinflammation, gut–brain axis

## Abstract

Huntington’s disease (HD) is a rare but progressive and devastating neurodegenerative disease characterized by involuntary movements, cognitive decline, executive dysfunction, and neuropsychiatric conditions such as anxiety and depression. It follows an autosomal dominant inheritance pattern. Thus, a child who has a parent with the mutated huntingtin (*mHTT*) gene has a 50% chance of developing the disease. Since the HTT protein is involved in many critical cellular processes, including neurogenesis, brain development, energy metabolism, transcriptional regulation, synaptic activity, vesicle trafficking, cell signaling, and autophagy, its aberrant aggregates lead to the disruption of numerous cellular pathways and neurodegeneration. Essential heavy metals are vital at low concentrations; however, at higher concentrations, they can exacerbate HD by disrupting glial–neuronal communication and/or causing dysbiosis (disturbance in the gut microbiota, GM), both of which can lead to neuroinflammation and further neurodegeneration. Here, we discuss in detail the interactions of iron, manganese, and copper with glial–neuron communication and GM and indicate how this knowledge may pave the way for the development of a new generation of disease-modifying therapies in HD.

## 1. Introduction

Huntington’s disease (HD) is a relentlessly progressive and debilitating adult-onset neurodegenerative disorder characterized by a well-defined clinical triad: motor dysfunction including choreoathetosis (involuntary twitching, twisting or squirming movements), where severe cases can cause permanent disability, cognitive decline including memory impairment and executive dysfunction, and psychiatric disturbances including anxiety and depression [1,2]. Pneumonia, followed by cardiovascular diseases, are the common causes of death [1,3]. Suicide is also more prevalent in patients with HD compared to the general population [1].

HD is an autosomal dominant disease with characteristic cytosine, adenine, and guanine (CAG) trinucleotide repeats on the short arm of chromosome 4p16.3 within the huntingtin (*HTT*) gene, leading to the production of a mutant huntingtin protein (mHTT) [2,4]. HTT is involved in many critical cellular processes including neurogenesis and brain development, energy metabolism, transcriptional regulation, synaptic activity, vesicle trafficking, cell signaling, and autophagy [2,4]. It is not surprising, therefore, that aberrant aggregates of this protein lead to the disruption of numerous cellular pathways, triggering a cascade of neurodegeneration [1,2,4].

HD is a rare neurodegenerative disorder (about 4 per 10,000 worldwide) with lower prevalence in Asia and higher prevalence in Europe, North America, and Australia, possibly due to the *HTT* gene haplotypes [2,5,6]. It typically manifests in mid-life, between the ages of 30 and 50 years, but can occur even before the age of 20, where it is termed juvenile HD. Diagnosis is made based on motor, cognitive, and behavioral tests and is confirmed via genetic testing using DNA analysis. Since no cure is available, treatment is aimed at improving the quality of life and decreasing complications.

Recent advances in molecular biology, focusing not only on the cellular pathways dysregulated by mHTT but also exploring the potential influence of external factors such as heavy metal exposure and gut microbiota (GM), have paved the way for the development of a new generation of disease-modifying therapies (DMTs) for HD [7,8]. In this review, following a brief description of HD pathology, we explore the roles of heavy metals in its etiology and focus on the potential manipulation of the GM as a novel therapeutic strategy.

## 2. HD Pathophysiology

Three significant categories of risk factors associated with CAG repeats have been identified in HD. The first and foremost is the length of the repeat, as the longer the repeats (>35), the earlier the onset of symptoms. Indeed, abnormal CAG triplet repeats lead to an abnormally elongated polyglutamine (polyQ) tract, which results in neurodegenerative diseases including HD [9]. CAG length is also a significant factor for the progression of the disease, especially in cognitive, motor, and neurological disturbances. The CAG repeats not only provide information on the age of clinical onset but also predict the age of death, as the course of the disease commonly lasts 15 to 20 years [1]. Second is the instability of CAG, and the third are the genetic modifiers that play an essential role in the progression of the disease [1]. The primary pathophysiological features of HD are the degeneration of neurons in the caudate, putamen, and cerebral cortex. The brain, particularly in the striatum, atrophies, showing extensive neuronal loss. It is believed that the choreiform movements and the development of dystonia and akinesia are due to the degeneration and loss of substance-P in the medium spiny neurons of the basal ganglia, and cognitive and behavioral dysfunctions are due to cortical atrophy [1]. Several theories have been suggested as reasons for pathogenesis. These include the accumulation of mHTT aggregates, leading to an impairment of the ubiquitin–proteosome pathway, transcriptional dysregulation, excitotoxicity due to increased release of glutamate and glutamate agonist from the cortical afferents, mitochondrial dysfunction and altered energy metabolism, changes in axonal transport, and synaptic dysfunction [1,10]. These contentions are because HTT is essential not only for the embryonic brain development but also for the adult brain function. Furthermore, mHTT may cause a gain of function or toxicity, or loss of function, either of which can contribute to the HD pathology [11].

mHTT is also a strong activator of glial cells, the brain’s immune cells, leading to chronic neuroinflammation [12]. While the initial activation of the glia is for neuroprotection, the overstimulation of these cells results in a neuroinflammatory response, which can cause neuronal damage and/or cell death, hence contributing to disease progression [12,13]. Recent research suggests the potential contributions of environmental factors like heavy metals such as iron (Fe), manganese (Mn), and copper (Cu) to HD pathology [12,13,14]. Heavy metal exposure further disrupts post-transcriptional mechanisms, exacerbating the problems caused by mHTT and decreasing the clearance rate of misfolded proteins, hence creating a vicious cycle that accelerates the neurodegeneration process [15]. Heavy metals may also indirectly influence neuroinflammation and/or mHTT clearance, causing further damage via their interaction with the GM, as discussed in more detail below.

The characteristic involuntary movements are progressive as they initially begin in the distal extremities and gradually move to proximal and axial muscles with greater amplitude and could extend to facial muscles. Whereas in the early stages, the symptoms manifest as hyperkinetic with involuntary chorea, in later stages, hypokinesia and dystonia predominate. In the later stages of the disease, the patient becomes bedridden due to severe rigidity and contractures in the extremities. Dysarthria and dysphagia and trouble in speaking and swallowing, respectively, develop during the course of the disease, which could lead to aspiration and pneumonia, the main cause of death in HD. Dystonia, characterized by increased muscle tone with slower movements, leads to abnormal posturing such as torticollis (stiff neck) and is usually one of the early signs of motor involvement in HD. Tics and ataxia may also develop. The progression of motor disturbances over time can lead to difficulties in walking and standing and frequent falls [1].

In addition to the motor symptoms, behavioral and cognitive disturbances manifest early on. Thus, initially, patients may present with impulsivity, poor attention, and irritability, leading to outbursts of anger and aggression. Later, emotional blandness with prominent apathy, loss of intuition, and creativity ensues. These are likely due to degeneration in the fronto-striatal pathway. Apathy, which is also progressive, is the most common feature of the disease. Mood disorders including depression are also common, which can lead to suicide in HD. Psychosis and cognitive decline to the point of unawareness appear later. Cognitive decline usually manifests before the onset of motor disturbances. The prominent cognitive changes include difficulty in planning, organizing, and multitasking, which may progress to dementia. Interestingly, it is believed that memory loss in HD is due to an inefficient search of memory (subcortical in nature) rather than a deficient in memory formation. In addition, more common features of cortical dementia such as apraxia and aphasia (speech disorders) are avoided in HD. Nonetheless, there is severe slowness in the psychomotor processes [1].

## 3. Current and Prospective Treatments

Beyond symptom management with dopaminergic and other medications [1,16,17], evolving therapeutics for HD target the molecular aspects with the intention of developing disease-modifying drugs [2,18]. These techniques include direct DNA/gene therapies to manipulate the *HTT* gene and correct the CAG repeat [19]. Thus, the potential of genome editing such as zinc-finger nucleases (ZFNs), transcription activator-like effector nucleases (TALENs), and the CRISPR/Cas9 system have been suggested [20]. RNA modulation may also be a promising approach, and antisense oligonucleotide (ASO) therapies and RNA interference (RNAi) therapies are currently undergoing clinical trials [16,20]. However, using ASOs to lower HTT by targeting transcripts has not been successful in human clinical trials [21]. Although gene therapy might be a promising future intervention, treatments addressing the functional aspects of HTT could be incorporated into current HD therapies. Attempts at enhancing neurogenesis are also being considered [2]. This is because HTT promotes BDNF expression and enhances BDNF vesicular trafficking along microtubules, and mHTT dysregulates these functions by suppressing BDNF transcription, resulting in lower central BDNF levels [22,23,24]. Indeed, it has been suggested that BDNF may serve as a gauge in detecting the severity of HD [25]. Thus, BDNF provides an attractive target for pharmacotherapeutial developments in HD [24]. In addition, targeting mHTT, therapies using potent small molecules, ubiquitin proteasome, or the autophagy-lysosomal systems are also under consideration [26,27].

Other disease-modifying therapies target aberrant downstream pathways such as excitotoxicity, mitochondrial dysfunction, and neuroinflammation [1].

Excitotoxicity, due to an imbalance between excitatory and inhibitory neurotransmitters, has been a subject of intense studies for more than two decades [28,29]. Excessive stimulation by glutamate, the excitatory neurotransmitter, can result in cell death via calcium-mediated mitochondrial dysfunction. The increased cytoplasmic calcium directly targets the mitochondria and alters its membrane potential. This compromises the electron transport chain, a vital pathway for energy production within mitochondria. Consequently, the cell experiences reduced ATP synthesis, hindering its ability to maintain essential cellular functions. Furthermore, the mitochondrial dysfunction compromises the antioxidative processes and leads to the overproduction of reactive oxygen species (ROS) [16,20]. Calcium dysregulation also activates apoptotic cell death pathways involving caspase-9 and caspase-3, accelerating programmed cell death [30].

Therefore, targeting the Glutamate/GABA imbalance may be a viable option in addressing some HD symptoms. This contention is further supported by the presence of aberrant NMDA receptor distribution in HD pathogenesis. Specifically, a reduction in palmitoylation, a post-translational modification, was observed in striatal NR2B-containing NMDA receptors of YAC128 mice, a model of HD. This decrease in palmitoylation correlated with an increase in extrasynaptic NMDA receptors, signifying a potential mislocalization of these receptors away from their typical synaptic sites, hence contributing to the vulnerability of striatal neurons in HD. These findings highlight the potential of targeting NMDA receptor palmitoylation as a therapeutic strategy for HD [29]. Medications like memantine and amantadine, both NMDA receptor antagonists, have shown effectiveness in at least the motor symptoms in HD [16,31,32].

As mentioned above, glial cells in general, and micro- and astroglia in particular, are major contributory cells to neuroinflammation, which is one of the aberrant pathways involved in the pathophysiology of HD [33,34]. Hence, below, we briefly discuss the potential role of glial cells in HD pathology.

## 4. Glial Cells—HD

Glial cells, outnumbering the neurons by 10 to 1, were once considered only to be structural support for the neurons. However, they are involved in numerous critical brain functions, including myelination, the formation of the blood–brain barrier (BBB), the development and remodeling of synapses, energetic support for neurons, the control of metabolism, the regulation of neurotransmitters and neuroendocrine function, the control of the fluid/electrolyte homeostasis, detoxification, and immune response [35]. Their dysregulation has been associated with neuropsychiatric and neurodegenerative diseases including HD [2,13,36,37]. Recently, we proposed that glial nAChRs may be a suitable target for intervention in Parkinson’s disease (PD) [38]. It would be of interest to determine if this hypothesis can extend to HD.

Four major glial cells (microglia, astrocytes, oligodendrocytes and synantocytes or NG2 cells) have been identified to date. We briefly discuss each with their relevance to neurodegenerative diseases in general, and HD in particular. Moreover, heavy metal interactions with these cells directly or indirectly via GM are also touched upon.

### 4.1. Microglia—HD

Microglia, constituting 10–15% of all central nervous system (CNS) cells, are considered the resident immune cells, as they constantly survey the environment and react quickly to any kind of insult. They play a vital role in maintaining homeostasis in the brain; however, their overactivation leads to neuroinflammation, which, as alluded to above, may be responsible for the manifestation of neuropsychiatric and/or neurodegenerative diseases [35]. Microglia also regulate the number of neuronal precursor cells, neurogenesis, and the formation and elimination of neuronal synapse and mediate infiltration of T cells into the brain [39].

Depending on the status of their activity, microglia are referred to as resting, activated, or phagocytic. Whereas at the resting or inactive state, they are highly ramified, when activated, they contract, assume an enlarged cell body, and proliferate. This happens in response to injury or insult, allowing them to carry their phagocytic activity, whereby debris is eliminated, and repair and recovery can ensue. This essential function can become detrimental if microglia are overactivated, causing neuroinflammation, followed by neurological anomalies [40,41,42,43].

Microglia express various receptors such as the calcium-sensing receptor (CASR), low-density lipoprotein receptor-related protein 1 (LRP1), triggering receptor expressed on myeloid cells-2 (TREM2), nicotinic cholinergic receptors (nAChRs), and toll-like receptors 2 and 4 (TLR2 and TLR4) [43]. TLRs are the subject of intense investigation as potential targets for neuropsychiatric/neurodegenerative diseases as they facilitate the removal of debris or pathogens by initiating the innate immune response [39,44,45,46].

Importantly, heavy metals (discussed in detail below) can activate microglia and trigger neuroinflammation and neuronal death [47].

### 4.2. Astroglia (Astrocytes)—HD

Astroglia, or astrocytes, have a wide distribution in the brain and may constitute up to 60% of the total cells in certain areas of the brain. They provide nutrients for the neurons, remove waste, monitor and regulate pH homeostasis, and are key components of the BBB. Moreover, they have extensive synaptic connections with the neurons and help to maintain neuronal integrity [39,48,49,50]. They are also key mediators of excitotoxic glutamate reuptake [51,52]. More recently, it was reported that astrocytes are the necessary source of TNF-α for the mediation of homeostatic synaptic plasticity [53]. Astrocytes contain their own neurotrophic factor, referred to as glial cell line-derived neurotrophic factor (GDNF), a protein that, like brain-derived neurotrophic factor (BDNF), provides trophic support for the growth and differentiation of synapses and promotes cell survival [49,53]. Astrocytes also express high levels of glial fibrillary astrocytic protein (GFAP), which is important for astrocyte–neuron communication, and helps to maintain the mechanical strength, shape, and movement of the cell and is commonly used as a marker for their identification [53,54].

Interestingly, astrocytes can become reactive by polarized microglia to help with defense mechanisms and the removal of pathogens [39]. However, also in this case, the overstimulation of these cells will result in the production of pro-inflammatory cytokines and contribute synergistically to neuronal dysregulation and/or death [55,56,57]. In this regard, heavy metals can cause astrocyte dysfunction, triggering neuronal, as well as oligodendrocyte, malfunction [58,59,60]. Moreover, as the BBB controls the transport of nutrients and metabolites into the brain and limits the access of harmful substances, its disruption is associated with the pathophysiology of major neurological disorders. For example, lead-induced damage of the BBB has been implicated in autism spectrum disorder (ASD), whereas Cu, Mn, and Fe disruption of the BBB have been linked to HD [14,61,62]. Finally, the GM, which, via short-chain fatty acids (SCFAs), maintains the integrity of the BBB, may be highly impacted by gut dysbiosis (discussed below).

### 4.3. Oligodendrocytes—HD

Oligodendrocytes (OLs), constituting 75% of all glial cells, are well recognized as the primary source of myelination in the CNS [63]. They control extracellular potassium concentration, modulate axonal growth, provide metabolic and trophic supply to myelin, secrete GDNF and BDNF, and, like microglia and astrocytes, express TLRs, which are also necessary for myelin formation [64,65,66]. Myelinated axons, which comprise the white matter, connect various gray matter areas (consisting of neuronal bodies, axon terminals, and dendrites) of the brain to each other and carry nerve impulses between neurons. Abnormality in white matter has been considered as an early indicator in HD [67]. Importantly, heavy metals can cause dysfunction in these cells as well [58,59,68].

### 4.4. Synantocytes (NG2 Cells)—HD

The fourth subset of major glial cells in CNS, synantocytes, are OL-precursor cells that are almost uniformly distributed in both white and gray matter areas, associate closely with neuronal cell bodies and dendrites, and maintain the ability to keep proliferating in the adult brain [63,69,70]. These cells can also give rise to astrocytes and neurons [63,69,70], and their potential involvement in neurodegenerative diseases is suspected [71,72]. For example, neuroinflammation and increased BBB permeability in experimental autoimmune encephalomyelitis (EAE) have been attributed to NG2 cells in [73], where it was postulated that NG2 cells, via the stimulation of reactive T cells, control IL-12 expression [73]. NG2 cells have been implicated in neuroinflammation [74] and neurovascular unit formation during development [75]. Following acute ischemic stroke, NG2 cells play a key role in angiogenesis and the generation of OLs [75]. Because of their influence on neuronal plasticity and communication with neurons, OLs may provide a novel target for therapeutic interventions in a variety of neurological diseases [75,76,77]. Whether heavy metals interact with NG2 cells is yet to be determined.

## 5. Gut Microbiota

GM is a complex and dynamic population of trillions of bacteria, fungi, archaea, and eukarya found in the gastrointestinal tract (GI). Microbiome refers to the genetic composition of these cells, which is now estimated to be slightly higher than the human genome [78,79,80]. GM exhibits remarkable diversity that changes over a person’s lifespan following a symbiotic relationship with the host. It plays a vital role in brain development, digestion, nutrient absorption, the fermentation of undigested carbohydrates, the production of essential vitamins and metabolites like SCFAs, the regulation of the immune system, the maintenance of BBB integrity, and overall health [81,82]. Dysbiosis, referring to an imbalance in the composition and function of the GM, has been implicated in a wide range of pathological processes, including digestive, metabolic, autoimmune, and neurological disorders [62,83,84].

The immune system plays a major role in the perpetuation and maintenance of the symbiotic relationship between the host and the beneficial commensal bacterial strains. Due to its substantial influence on physiological processes, as well as its wide implication in various pathological states, the GM is considered to be a new ‘metabolic organ’, having a major influence not only on the digestive system but also on other organs, notably the CNS [8,83,85].

## 6. Gut–Brain Axis

A bidirectional communication pathway, termed the gut–brain axis (GBA), that links the GM to the CNS is well recognized [86]. This axis facilitates communication through the vagus nerve, the immune system, and microbial metabolites. Dysbiosis has been increasingly implicated in the pathogenesis of various neurological disorders through several mechanisms, the most prominent being the neuroinflammation. In dysbiosis, there is the release of pro-inflammatory mediators such as cytokines (e.g., interleukin-1β, tumor necrosis factor-α) and chemokines from the immune cells, which can then migrate to the CNS via the bloodstream or lymphatic system and exacerbate neuroinflammation, affecting brain development and behavior [84,87,88,89].

Some of the metabolites produced by the GM, such as the SCFA butyrate, contribute to epithelial defense and have antioxidant and anti-inflammatory properties [90,91]. Some other metabolites such as lipopolysaccharides (LPSs) are pro-inflammatory and used to mimic inflammatory diseases [92,93]. An imbalance in the GM may also weaken the intestinal barrier, allowing bacterial products and toxins to translocate into the bloodstream. This phenomenon, known as the leaky gut, highlights the significance of maintaining the integrity of the GM [93,94]. Of direct relevance to the topic of our discussion are the recent reports implicating dysbiosis in HD, which is elaborated below [84,95].

## 7. Heavy Metals

Heavy metals are essential for a variety of biological functions [38,96,97,98]. For example, iron (Fe) is a critical component of many vital enzymes or coenzymes such as catalases and cytochromes, which mediate cellular processes and drug metabolism. Indeed, catalases, by neutralizing hydrogen peroxide, are critical in providing protection against oxidative stress [38]. Fe is also an essential component of hemoglobin, where its deficiency leads to Fe-deficiency anemia [99]. Similarly, Mn acts as an activator or cofactor for a variety of metalloenzymes that are essential for normal cell growth and development [100,101,102,103,104]. Moreover, the enzymes or coenzymes utilizing Mn play key roles in functions such as gluconeogenesis, the suppression of oxidative stress (Mn superoxide dismutase, SOD) and conversion of glutamate into glutamine (glutamine synthetase) [105,106], all of which have critical biological functions. Copper (Cu) is another metal essential for the synthesis of red blood cells, collagen, bone, and connective tissue and maintenance of nerve cells and the immune system. It is required for adequate growth, cardiovascular integrity, lung elasticity, neovascularization, neuroendocrine function, and Fe metabolism [98,106]. However, at higher concentrations, it can contribute to HD pathology. Below, we discuss the relevance of each essential heavy metal to HD vis-à-vis their interaction with GM and inflammatory processes.

### 7.1. Iron (Fe)—HD

Ferroptosis is a newly discovered form of programmed cell death distinct from apoptosis and necrosis. It is considered a key contributor to the pathogenesis of neurodegenerative diseases [107,108]. This section focuses on evidence linking it to neurodegeneration, particularly to HD [109].

Ferroptosis, an Fe-dependent form of regulated cell death, is characterized by the excessive peroxidation of polyunsaturated fatty acids (PUFAs) found within cell membranes. Enzymes like acyl-CoA synthetase long-chain family member 4 (ACSL4) and lysophosphatidylcholine acyltransferase 3 (LPCAT3) are key in the catalyzation of these reactions [110]. Mitochondrial dysfunction is a hallmark of ferroptosis and a necessary condition for the perpetuation of these reactions. In fact, increased Fe uptake promotes the generation of destructive hydroxyl radicals through the Fenton reaction, thus perpetuating the chain reaction of lipid peroxidation, ultimately compromising membrane integrity [111], which leads to cell membrane disruption and cell death. Unlike apoptosis and necrosis, ferroptosis presents with its own cellular pathways. While mitochondria are central to the execution of ferroptosis, other organelles contribute through stress-related pathways. The endoplasmic reticulum, Golgi apparatus, and lysosomes can be involved in amplifying the cell death program [112,113].

Ferroptosis is an oxidative process that needs to be controlled via counter regulatory mechanisms. In this context, glutathione, a major antioxidant system in conjunction with its key enzyme glutathione peroxidase 4 (GPX4), plays a major role in protecting cells from uncontrolled ferroptosis by suppressing lipid peroxidation [114]. Fe metabolism, cysteine availability, and lipid homeostasis are tightly intertwined and serve as key regulatory points for ferroptosis induction or inhibition. Unregulated ferroptosis is a major determinant of neuroinflammation and neurodegenerative diseases [109].

Regarding HD, it has been shown that the aggregation and accumulation of mHTT increases the susceptibility of basal ganglia neurons to ferroptotic cell death [115]. Fe overload not only disrupts mitochondrial functions, leading to impaired energy production and increased oxidative stress, but also generates highly reactive free radicals that damage lipids, proteins, and DNA and disrupt the redox balance making neurons more susceptible to ferroptotic cell death, thus exacerbating HD [14,116,117,118]. Indeed, elevated levels of lipid peroxidation products have been detected in both cellular HD models and HD patients [119].

A direct interaction between Fe and mHTT is also evident whereby Fe enhances mHTT aggregation and its neurotoxic effect. This creates a vicious cycle that accelerates neurodegeneration [27,120]. In the same way, mHTT might interfere with System Xc-, an antiporter that exchanges glutamate (excitatory neurotransmitter) for cystine (precursor for glutathione synthesis), leading to decreased glutathione (GSH) levels, a crucial antioxidant that protects cells from ferroptosis [27,120].

It is worth noting that alterations in the GM composition and/or function could impact the absorption and metabolism of dietary Fe in the GI tract. Moreover, changes in Fe metabolism may, in turn, affect Fe levels in the brain and contribute to the neurodegenerative process in HD [121,122,123].

Based on these findings, several therapeutic perspectives have been explored. Among them are the ferroptosis inhibitors and Fe chelators, which have shown promising neuroprotective effects in HD models [16,124,125,126].

### 7.2. Manganese (Mn)—HD

Another essential heavy metal implicated in HD pathophysiology is Mn. As alluded to earlier, Mn is a crucial cofactor for many enzymes and is necessary for amino acid, cholesterol, glucose, and carbohydrate metabolism; reactive oxygen species scavenging; bone formation; reproduction; and the immune response [127,128]. Mn deficiency can lead to weakness, seizures, infertility, and bone malformation. Mn overload, on the other hand, concentrates in the brain, especially in the basal ganglia, resulting in Parkinsonism [129]. Early life exposure to high levels of Mn is thought to impact neurodevelopment, especially cognitive behavior in children [130]. Importantly, high Mn exposure and alteration in the GM has been linked to oxidative stress and neuroinflammation, which are implicated in HD [131,132].

The potential link between Mn, insulin/IGF signaling, and HD, whereby Mn deficiency was shown to share cellular consequences such as increased oxidative stress and mitochondrial dysfunction with HD, was reviewed recently [133]. It was concluded that Mn can mimic some actions of insulin/IGF signaling in HD models, thereby providing protection in instances where HD symptoms might be precipitated by Mn deficiency [133].

### 7.3. Copper (Cu)—HD

Cu toxicity has also been linked to HD [134]. Cu, as mentioned earlier, is an essential metal that plays a critical role in various neurochemical processes, where its dysregulation is detrimental [135]. Studies highlight its potential contribution to neurodegeneration in HD via the enhancement of mHTT toxicity [15]. Cu may also disrupt proteostasis, the process of protein folding and degradation, further contributing to cellular dysfunction [136]. It is noteworthy that Wilson’s disease also involves the disruption of Cu metabolism and its deposition in the basal ganglia. People suffering from this genetic disorder present with extrapyramidal signs and symptoms ranging from movement disorders (tremor, dystonia, parkinsonism) to cognitive and speech impairment and psychiatric symptoms, similar to what is observed in HD [137]. Thus, like Fe, Cu may promote mHTT aggregation and toxicity. Cu also modulates the interaction between huntingtin inclusions and the autophagy adaptor protein, which is responsible for the clearance of the toxic aggregate [15,138,139,140]. A study using the drosophila model of HD showed that D-penicillamine, a Cu chelator, significantly reduced the formation of amyloid-like huntingtin aggregates, suggesting a potential therapeutic avenue for mitigating the toxicity associated with huntingtin aggregation [15].

In summary, epidemiological and clinical studies have shown a strong correlation between aberrant metal exposure and several neurological diseases, including HD [141]. For example, toxic effects of long-term exposure to copper, zinc, and their mixture, in a C. elegans-based HD model, was recently reported [134]. Similarly, cadmium, Fe, Mn, and Cu have been implicated [141,142,143]. Thus, it would be of significant clinical relevance to investigate whether the prevalence of HD correlates with exposure to high levels of heavy metals in select populations.

## 8. Heavy Metals and GM—HD

Building upon the intriguing link between heavy metal dysregulation and HD, recent research is exploring the potential influence of the GM in HD pathophysiology. The GM is in fact increasingly recognized for its role in brain health and disease [93,95]. The GM can both influence and be influenced by heavy metal exposure. Certain gut bacteria can facilitate the absorption and accumulation of heavy metals like Fe, lead (Pb), and Cu in the body [62]. Conversely, heavy metal exposure can disrupt the composition and function of the GM, triggering inflammatory responses that can indirectly impact the basal ganglia and exacerbate HD pathology [144]. The basal ganglia, a control center for movement, cognition, and emotional regulation, is critically affected in HD and is particularly susceptible to GM-derived neuroinflammation [144]. Thus, by promoting a healthy GM composition through dietary interventions or prebiotics/probiotics, the absorption of heavy metals like Fe and Cu may be curtailed, thereby mitigating their potential contribution to HD pathology.

The intricate relationship between the GM and HD pathophysiology is a burgeoning area of research with significant therapeutic potential. Recent studies suggest a multifaceted interplay between gut bacteria, the immune system, and the CNS that may contribute to HD progression [84,145,146,147]. One key mechanism in this scenario involves SCFAs produced by beneficial bacteria like *Bifidobacterium* and *Faecalibacterium prausnitzii* that exert neuroprotective effects [62,148,149]. Thus, in a mouse model of HD, SCFA supplementation can improve motor function, reduce mHTT aggregation, and mitigate neuroinflammation. Conversely, dysbiosis, leading to LPS production, has been linked to an increase in mHTT aggregation and neuronal death [150].

Another critical link in GBA is a bidirectional communication pathway involving the vagus nerve, immune signaling, and the production of neurotransmitters. Dysbiosis can trigger chronic low-grade inflammation in the gut, leading to the activation of immune cells and the release of pro-inflammatory cytokines. These inflammatory signals can then travel up to the brain via the vagus nerve, promoting neuroinflammation and further compromising neuronal health in the basal ganglia [151]. Interestingly, mHTT was shown to be widely expressed in the intestines, which would allow it to interact with the GM, hence affecting the progression of HD [146]. GM involvement in HD pathology has also been verified in several animal models [145,152].

It was mentioned earlier that a leaky BBB, characterized by increased permeability, allows the passage of harmful bacterial products and inflammatory molecules into the brain. It is noteworthy that dysbiosis can also disrupt the tight junctions of the BBB, potentially accelerating neurodegeneration in HD [153].

## 9. Conclusions

**Figure 1:**
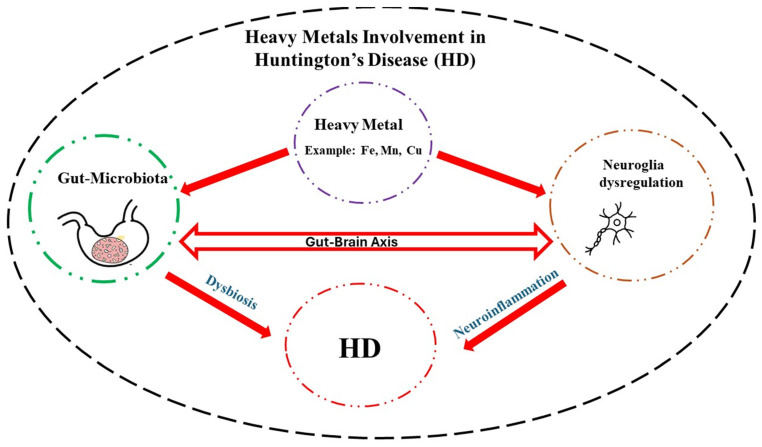
Schematic diagram depicting how heavy metals, via their interactions with the neuroglia and gut microbiota, may contribute to Huntington’s disease (HD) pathology. A detailed understanding of this interaction can pave the way for novel therapeutic interventions in this rare but devastating neurological disease.

Neurodegenerative diseases exact a tremendous toll on those affected and their caregivers. Although, in most cases, the etiology is unknown, in the case of HD, a mutation in huntingtin gene is the main culprit. In this regard, efforts are underway to quantify the mutant protein in the cerebrospinal fluid with the aim of developing effective therapies [154]. In addition, exposure to high levels of essential heavy metals such as Fe, Mn, and Cu may exacerbate HD symptoms by disrupting neuronal communications, particularly glia–neuron interaction. High levels of heavy metals, via their interaction with the GM and induction of dysbiosis, can also promote neuroinflammation and, hence, indirectly contribute to HD pathology (Figure 1). Understanding the intricate coordination of the GBA and specific effects of each heavy metal on this axis may provide further therapeutic intervention in this devastating disease [8,155,156].
